# Label free tracking to quantify nanoparticle diffusion through biological media

**DOI:** 10.1038/s41598-024-69506-0

**Published:** 2024-08-13

**Authors:** Genevieve Schleyer, Eann A. Patterson, Judith M. Curran

**Affiliations:** 1 Department of Materials, Design & Manufacturing Engineering, University of Liverpool, Brownlow Hill, Liverpool, UK; 2 Department of Mechanical and Aerospace Engineering, University of Liverpool, Brownlow Hill, Liverpool, UK

**Keywords:** Biomedical engineering, Microscopy, Nanoparticles

## Abstract

Nanotechnology is a rapidly evolving field and has been extensively studied in biological applications. An understanding of the factors that influence nanoparticle diffusion in biofluids can aid in the development of diverse technologies. The development of real-time, label-free tracking technologies would allow the expansion of current knowledge of the diffusion and activity of nanoparticles. Fluorescence-based microscopy is one of the most widespread tools to monitor and track nanoparticle dynamics; however, the influence of fluorescent tags on diffusion and biological activity is still unclear. In this study, we experimentally determined the diffusion coefficient of gold nanoparticles using a label-free, optical tracking technique and evaluated the influence of protein concentration, charge and diameter on nanoparticle diffusion through biological media. We dispersed positively- and negatively-charged nanoparticles with diameters varying from 10 to 100 nm in a common cell culture media with different concentrations of serum proteins. Our results show that dynamic protein interactions influence nanoparticle diffusion in the range of serum concentrations tested. Experimental regimes to obtain quantitative information on the factors that influence the dynamics of nanoparticles in biological media have been developed.

## Introduction

Nanoparticles are being studied extensively for use in medical applications, for example, drug delivery, tumour destruction via heating, diagnostics and as replacements to fluorescent labels for bioanalysis^1,2^. The use of nanoparticles in these applications can improve disease diagnosis and treatment specificity^3^, improve the efficacy of drugs, biodistribution and limit their systemic side effects^4^. When dispersed in a biological medium, nanoparticles come in contact with complex mixtures of proteins and extracellular material that have been found to influence the physico-chemical properties of the nanoparticle, for example, through the formation of a protein corona. There is little understanding of how the presence of soft matter influences the dynamics of nanoparticles in a biological medium although several studies have characterised the composition and size of the protein corona under various conditions^5–12^. A recent study quantified the sedimentation behaviour of 100 nm diameter gold nanoparticles in cell culture media supplemented with fetal bovine serum (FBS)^13^ in an initial attempt to assess the dynamics of nanoparticles in biological solutions. It was found that there was no significant difference in the settling dynamics of nanoparticles in cell culture media compared to water. This was reported to be due to the masking of the initial particle charge by the formation of a protein corona preventing rapid aggregation. The design parameters for nanoparticle drug carriers and other nanoparticle therapeutics are vast, emphasising the need for further investigation into the dynamics of nanoparticles in biological mediums. Diffusion is one of the processes that regulate the motion and transport of particles dispersed in solution. The speed of diffusion contributes to the time taken for nanoparticles to reach a target site and influences the sedimentation rate of nanoparticles. Thus, understanding the factors that influence the dynamics of nanoparticles in biological solutions is key to the design, development and optimisation of nanoparticles intended to be dispersed in a physiological environment.

### Nanoparticle diffusion

A deep fundamental understanding of the forces and mechanisms underpinning the dynamics of entities exhibiting diffusive motion in biological environments is necessary to improve the performance of diverse technologies. Diffusion of sub-micron particles is described as a classical problem in fluid dynamics^14^. Diffusion of particles in a solution is driven by the force of many fluid molecules colliding with the immersed object. The resulting motion is known as Brownian motion, which is characterised by a chaotic, random walk^15^ that facilitates the distribution of nanoparticles in the body. The diffusion of micro and macro particles in a fluid can be predicted by the widely-accepted Stokes–Einstein relationship which considers the particle size and fluid viscosity. For particles at the nanoscale, the diffusion process is governed by microscopic parameters rather than the macroscopic parameters in the Stokes–Einstein relationship^16^. A study on particle diffusion has shown that the transition from classical Stokes–Einstein diffusion to molecular diffusion occurs at the nanoscale^17^, impacting the current knowledge of diffusion of nanoparticles and highlighting a demand for experimental methods to quantify nanoparticle dynamics. There are multiple limitations to past and current single-particle tracking techniques. Methods based on fluorescence microscopy are at risk of photobleaching and phototoxicity due to prolonged exposure of a fluorescent label to the light source. There is also little knowledge on how fluorescent labels influence nanoparticle dynamics^18^. Label-free tracking techniques, such as dark field microscopy^19^, photothermal imaging^20–22^, plasmon-resonance microscopy^23^ and differential interference contrast microscopy^24,25^ require highly complex optical arrangements or specialised equipment, limiting the accessibility of these techniques. This study reports on the use of a label-free, optical, single particle tracking method to quantify the diffusion of nanoparticles using a standard inverted optical microscope adjusted to produce near-coherent light to generate optical signatures of nanoparticles, or caustics, as described by Patterson and Whelan ^26^. The aim of the study is to provide accurate measurements of the diffusion coefficient of nanoparticles with various physical and chemical properties in solutions that mimic physiological environments.

## Materials and methods

### Nanoparticle tracking

**Figure 1:**
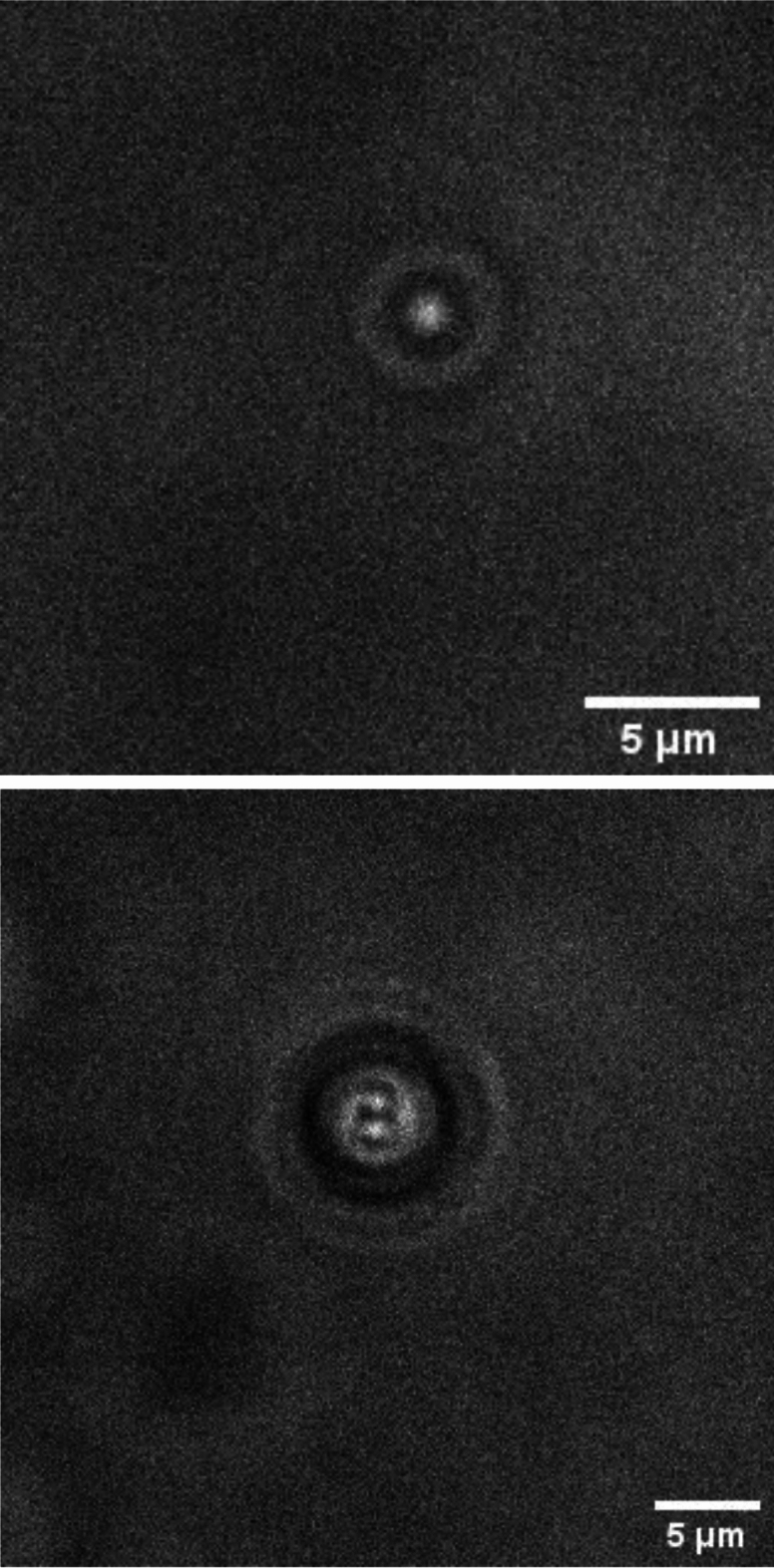
Caustic signature of 50 nm negatively-charged single nanoparticle (top) and aggregate (bottom) in 10% FBS DMEM.

Gold citrate stabilised nanoparticles were purchased (BBI Solutions, Crumlin, UK) in deionised water with diameters of 10nm (EM.GC10/4), 50nm (EM.GC50/4) and 100nm (EM.GC100/7). Zeta potentials were reported to be ~ -40mV by the manufacturer for all nanoparticle sizes. Positively-charged gold nanoparticles with branched polyethylenimine (BPEI) surfaces were purchased (Nanocomposix, San Diego, CA, USA) also with diameters of 10nm (AUBB10-1M), 50nm (AUBB50-1M) and 100nm (AUBB100-1M). The zeta potentials of these nanoparticles were characterised by the manufacturer and reported to be + 44.5 mV, + 63.8 mV and + 59.4 mV for the 10nm, 50nm and 100nm diameter particles, respectively. Both the positively- and negatively-charged nanoparticles were diluted from their as-supplied concentrations to concentrations in the range of 10^7^–10^9^ particles/ml. Nanoparticles were dispersed in Dulbecco’s Modified Eagle Medium (Gibco, Thermo Fisher, Waltham, MA, USA; cat. no. 12491015,) supplemented with fetal bovine serum (Sigma Aldrich, Saint Louis, MO, USA; cat. No. 12306C), filtered using a 0.2 μm syringe filter at concentrations of 2.5%, 5%, 10% and 25%. The concentration of nanoparticles was reduced by adding 100 μl of the nanoparticles in solution to 900 μl of cell culture media. This process was repeated until the desired working concentration of nanoparticles was reached. The samples were mixed to combine the nanoparticles with the media using a vortex mixer (Thermo Fisher) and then placed in a sonication bath for 5 min to separate loosely aggregated particles and ensure monodispersion of the nanoparticles in the sample. Caustic signatures of single particles can be easily distinguished between aggregated particles at the concentrations used in this study (Fig. 1). Immediately after, 50 μl were removed from the sample using a pipette and placed in a 250 μm microscope slide cavity (Eisco, Victor, NY, USA; cat. no. BI0086B) and covered with a glass cover slip (Camlab, Academy, Over, UK; cat. no. 13/2222). Nanoparticles were visualised using a standard inverted optical microscope (Axio Observer Z1, Carl Zeiss, Oberkochen, Germany) in transmission mode with some simple, reversible adjustments. The adjustable aperture in the condenser lens-assembly was reduced to its minimum (approx. 1 mm) and narrow band (550 nm) filter was inserted to increase the coherence of the light source which has been shown to increase the sharpness of the caustic signatures^27^. The microscope was set up for Kohler illumination^28^ and adjusted to be out-of-focus to allow the caustic signatures to be imaged. The microscope was equipped with an incubation stage (Incubator PM S1, Temp and CO_2_ module S1, Carl Zeiss, Oberkochen, Germany) which was set at 37 °C. A 40 × objective lens together with a camera supported by the ZEN software (Axiocam z1.m, Carl Zeiss, Oberkochen, Germany) were used to visualise and record videos of the nanoparticles. The videos of the nanoparticles were recorded for a period of 100 frames at 40 fps which corresponded to 2.5 s. This was found to be optimum for capturing the nanoparticle in the focus plane and was sufficient to give accurate values of the diffusion coefficient. For each combination of particle size, charge and FBS concentration four independent samples were prepared i.e. N = 4, in which six particles per sample were tracked. Averages of the diffusion coefficient were calculated which are presented in the figures below.

### Calculation for the experimental diffusion coefficient

The experimental diffusion coefficient for each particle was calculated using the mean square displacement which is a measure of the random steps made by an object in solution as previously described^17,29^. The technique used in this study allows the coordinates of the nanoparticle to be extracted frame-by-frame which are used to calculate the mean square displacement. In two dimensions the square displacement at the *i*th position is expressed by the equation below^30^.

1 $$(\Delta {r}_{i}{)}^{2}=(\Delta {x}_{i}(\Delta t){)}^{2}+(\Delta {y}_{i}(\Delta t){)}^{2}$$

The mean square displacement, $\langle (\Delta r{)}^{2}\rangle$ can then be used to determine the diffusion coefficient experimentally, as follows:

2 $$\langle (\Delta r{)}^{2}\rangle =dD\Delta t$$

where *d* is the number of dimensions, *D* is the diffusion coefficient and Δ*t* is the time between frames.

### Laser scanning confocal microscopy (LSCM)

Nanoparticles were prepared in the same way as described earlier. After the dispersion of nanoparticle in cell culture media, the samples were incubated at 37 °C for 30 min. NP-protein complexes were then isolated from excess media and serum by centrifugation for 5 min at a speed of 200 rpm. Media was then carefully removed using a pipette, leaving the concentrated nanoparticles at the bottom of the sample tube. The sample was re-dispersed in PBS and the centrifugation process repeated several times to remove excess and loosely bound proteins. 10 μl of concentrated nanoparticle solution was removed from the sample tube and placed on an adhesive carbon tab fixed to a 13 mm round cover slip which was left to dry in a 24-well plate. NP-protein complexes were blocked with 3% skimmed milk in PBS and left at room temperature for 1 h. The blocking solution was then removed and nanoparticle washed with PBS thoroughly. Anti-albumin (bovine serum) rabbit IgG fraction primary antibody (Thermo Fisher, Waltham, MA, USA; cat. no. A1113) was diluted 1:100 with 1% skimmed milk in PBS and applied to dried nanoparticles. BSA was chosen to be stained as it is the most abundant protein found in FBS (~ 60%). The sample was incubated for 1 h at 37 °C. Nanoparticles were then washed with PBS. Alexa Fluor 488 goat anti-rabbit IgG (Thermo Fisher, Waltham, MA, USA; cat. no. A11008) was diluted 1:100 with 1% skimmed milk in PBS and applied to the dried nanoparticles. The sample was incubated for 1 h at 37 °C before being washed again with PBS and fixed to a microscope slide. Samples were visualised using a 10 × air objective lens.

## Results and discussion

**Figure 2:**
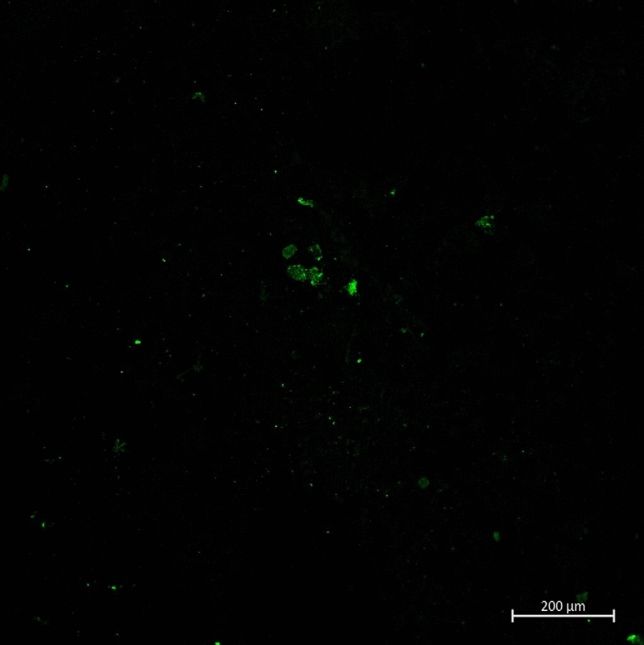
Laser scanning confocal microscopy images of bovine serum albumin (green) on 100 nm gold nanoparticles after incubation in 10% FBS DMEM for 30 min.

Understanding the factors that influence the diffusion of nanoparticles in biological fluids is of particular importance to drug delivery applications. Nanoparticles change their physical characteristics when exposed to a biological medium due to the instantaneous adsorption^31^ of proteins leading to the formation of a protein corona. The speed and quantity of protein adsorption has been linked with the physical characteristics of the nanomaterial such as size, charge and material^32^. The adsorption of proteins to a solid interface is a complex process, depending on relative abundance and affinity of protein molecules and an interaction between several adsorption subprocesses^33–35^. This paper does not deal with the different mechanisms of adsorption and qualitative differences in the protein corona, nor does it focus on changes in the nanoparticle physical characteristics. Rather, it presents experimental data demonstrating the influence of protein interactions on diffusion of nanoparticles in a commonly used cell culture media with typical FBS concentrations used in *in-vitro* testing. With the rise in nanoparticle uses in medicine, several studies have attempted to characterise the protein corona and its consequences on the physical and chemical properties of nanoparticles. Surprisingly, there is very little research on the influence of the protein corona on the dynamics of nanoparticles. Recent studies have highlighted the disparity between observed and predicted particle dynamics at the nanoscale making it clear that experimental methods that accurately quantify the diffusion of nanoparticles in biological media are needed and motivated this study. Figure 2 confirms that proteins were present after nanoparticles had been exposed to Dulbecco’s modified eagle medium (DMEM) supplemented with fetal bovine serum (FBS) and isolated from the media. The following sections discuss the influence of surface charge, serum concentration and particle size on nanoparticle dynamics in the biological medium.

### Time dependence of the protein corona and its influence on nanoparticle diffusion

It is well-known that protein corona formation on nanoparticles is a dynamic, time-dependent process^32^. Experiments carried out by Casals et al.^36^ showed the formation of a loosely bound ‘hard’ corona occurs over an incubation period of 5–30 min. During this time, proteins are in a continuous state of dynamic exchange with the incorporation of more stable proteins and removal of less stable ones. At longer time scales (up to 48 h), an irreversible ‘hard’ corona will form. As the nanoparticles in the current study were tracked within 5–30 min from the point of exposure to the cell culture medium and only for a few seconds (2.5 s per particle), it can be assumed that the nanoparticles experience dynamic adsorption and desorption of serum proteins which potentially influence the diffusion of nanoparticles.

### Influence of FBS concentration on nanoparticle diffusion

**Figure 3:**
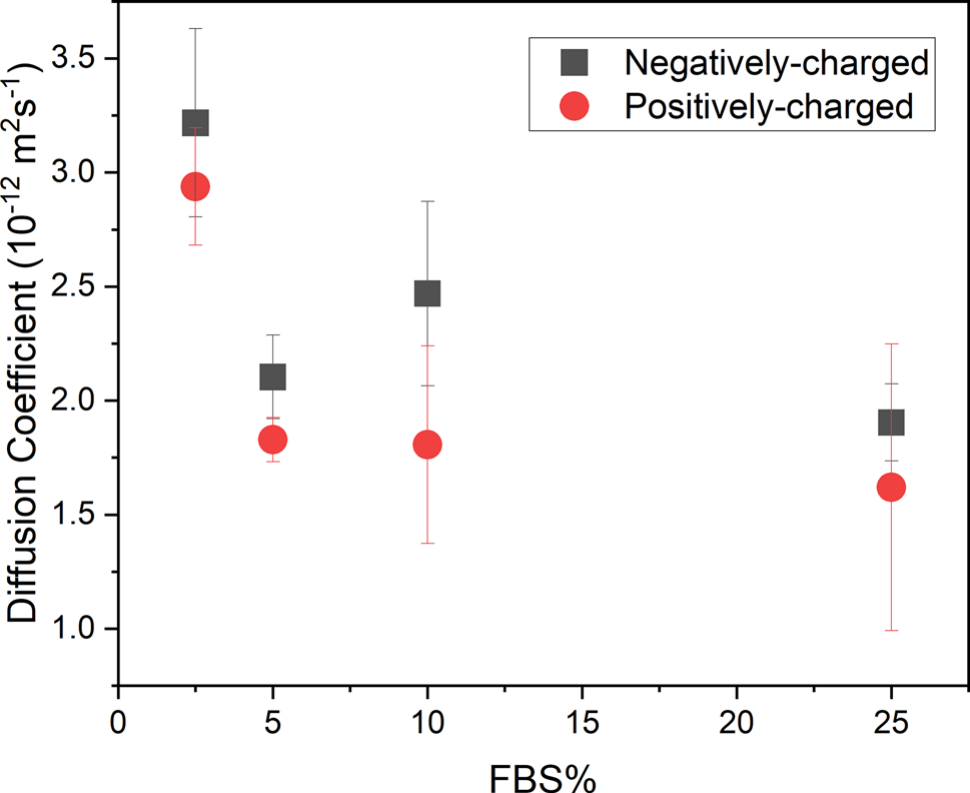
Average values (N = 4) of diffusion coefficient for negatively-charged (black squares) and positively-charged (red circles) nanoparticles with a diameter of 100 nm as a function of FBS concentration with error bars of ± 1 standard deviation.

The experimental diffusion coefficient of 100 nm gold nanoparticles in DMEM supplemented with different concentrations of FBS was calculated using Eq. (1) and the results shown in Fig. 3. The concentration of FBS does not influence the diffusion of 100 nm diameter nanoparticles except at 2.5% where the diffusion coefficient is significantly greater compared to particle diffusion in 5% FBS media for both positively- (p = 0.00040) and negatively-charged (*p* = 0.0036) nanoparticles. For similar particle concentrations, Coglitore et al.^17^ found that 100 nm diameter negatively-charged nanoparticles in simple media did not follow the Stokes–Einstein relationship but rather, could be described by the fractional Stokes–Einstein relationship. Their diffusion coefficient was the same for particles with diameters between 10 nm and about 150 nm. The data in Fig. 3 shows a change in the diffusion coefficient as FBS concentration increases. The adsorption of proteins resulting in an increase in particle size could be a contributary factor in shifting their behaviour into the Stokes–Einstein regime resulting in slower diffusion speeds for larger particles. Further testing found that the addition of FBS to DMEM above 2.5% does not sufficiently increase the viscosity of the medium to cause a reduction in the diffusion speed of the particles.

**Figure 4:**
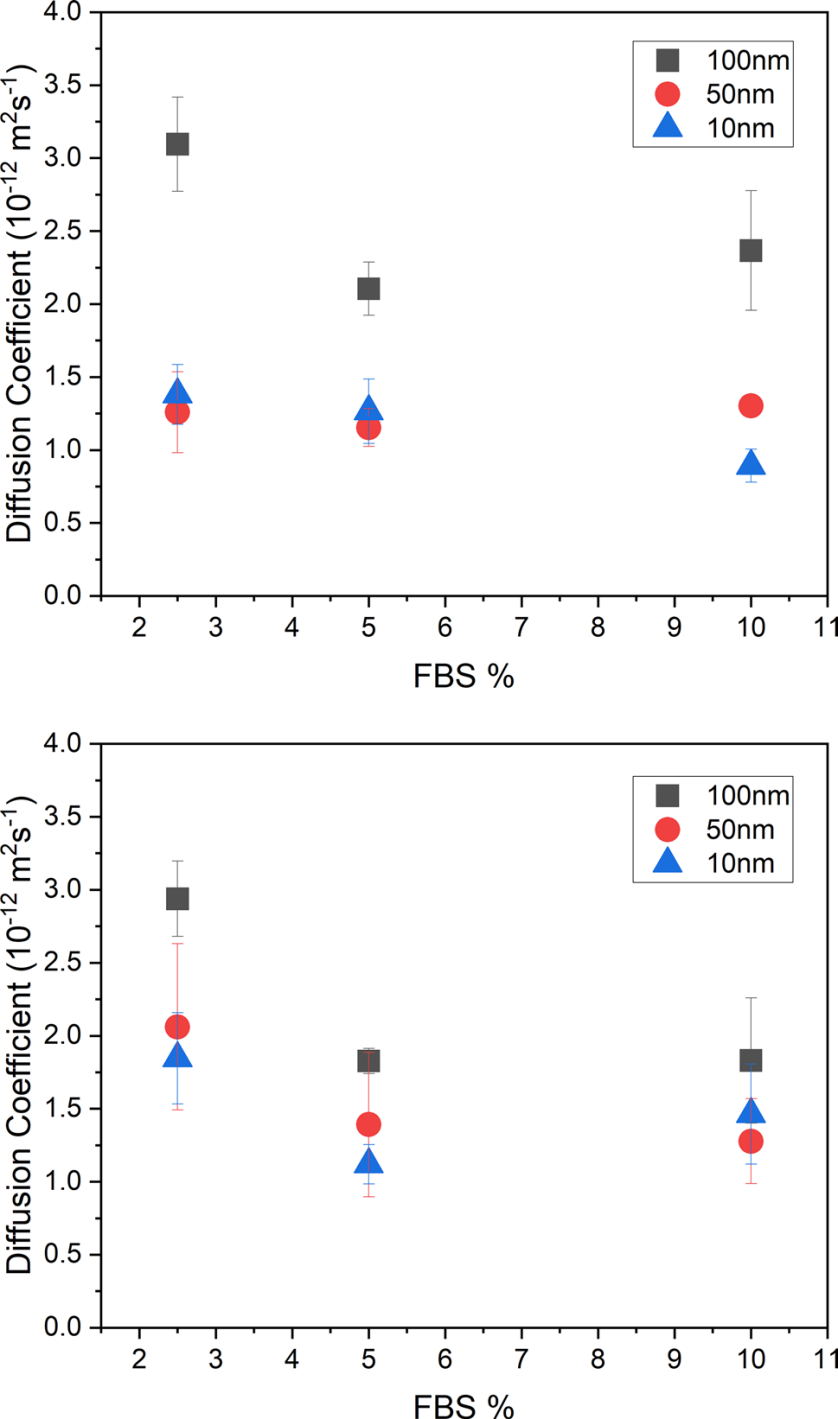
Average (N = 4) values of diffusion coefficient for 10 nm, 50 nm and 100 nm diameter negatively-charged (top) and positively-charged (bottom) gold nanoparticles in DMEM as a function of FBS concentration in the media with error bars of ± 1 standard deviation.

Gold nanoparticles with diameters of 10 nm and 50 nm were tracked in DMEM for three different concentrations of FBS and the results are shown in Fig. 4 with the results for 100 nm diameter particles at the same FBS concentrations. Similar to the 100 nm nanoparticles, positively-charged 10 nm nanoparticles show a significant decrease in the diffusion coefficient, *D* as FBS concentration increases from 2.5 to 5% (*p* = 0.010); however, negatively-charged 10 nm nanoparticles show a significant decrease as FBS concentration increases from 5 to 10% (*p* = 0.040). Both positively- and negatively-charged 50 nm nanoparticles show no change in the diffusion coefficient as FBS concentration increases. All positively charged nanoparticles display no change in the diffusion coefficient at concentrations higher than 5%, implying that these particles are still below the threshold at which the Stokes–Einstein relationship ceases to apply; in other words, any corona formed is not large enough to induce behaviour that follows the Stokes–Einstein relationship. It is clear from the literature that there are several factors that potentially could influence the dynamics of nanoparticles in cell culture media supplemented with FBS. For example, Davidson et al.^37^ investigated nanoparticle-protein interactions in different biological environments and found protein corona size significantly increases as protein concentration increases. Other studies have demonstrated that the protein corona thickness, density and composition can vary depending on the concentration of proteins in the media^5,10,11^. FBS is essential for the growth of many cell lines for tissue engineering applications; moreover, the data shows that some changes in the concentration of proteins in cell culture media affects nanoparticle diffusion. This was found to be primarily at lower concentrations of FBS. The specific effect of different protein concentrations on the dynamics of nanoparticles differs from particle-to-particle depending on size and charge, emphasizing the fact that universal rules about protein interactions and their influence on nanoparticle dynamics cannot be applied. For the majority of nanoparticles tested, at concentrations of FBS greater than 2.5%, the diffusion coefficient showed no significant change. This is further confirmed by the results for the diffusion of 100 nm nanoparticles in 25% FBS media. We recognise this is not a common concentration of FBS used in the culture of cells but demonstrates the insignificant change in diffusion coefficient as FBS concentration increases. This is the first study to show a direct correlation between diffusion speed and FBS concentration.

### Influence of charge on nanoparticle diffusion

Surface charge had no effect on the diffusion of 100 nm and 50 nm nanoparticles within the range of FBS concentrations tested (Fig. 3); however, at 10% FBS concentrations, charge had a significant effect on the 10 nm nanoparticles (*p* = 0.034). The type, quantity and kinetics of protein adsorption on nanoparticles is charge dependent as has been demonstrated by several researchers^12,38–40^. As a result, the difference between the dynamics of the larger positively- and negatively charged nanoparticles is more predictable compared to smaller nanoparticles.

### Influence of size on nanoparticle diffusion

**Figure 5:**
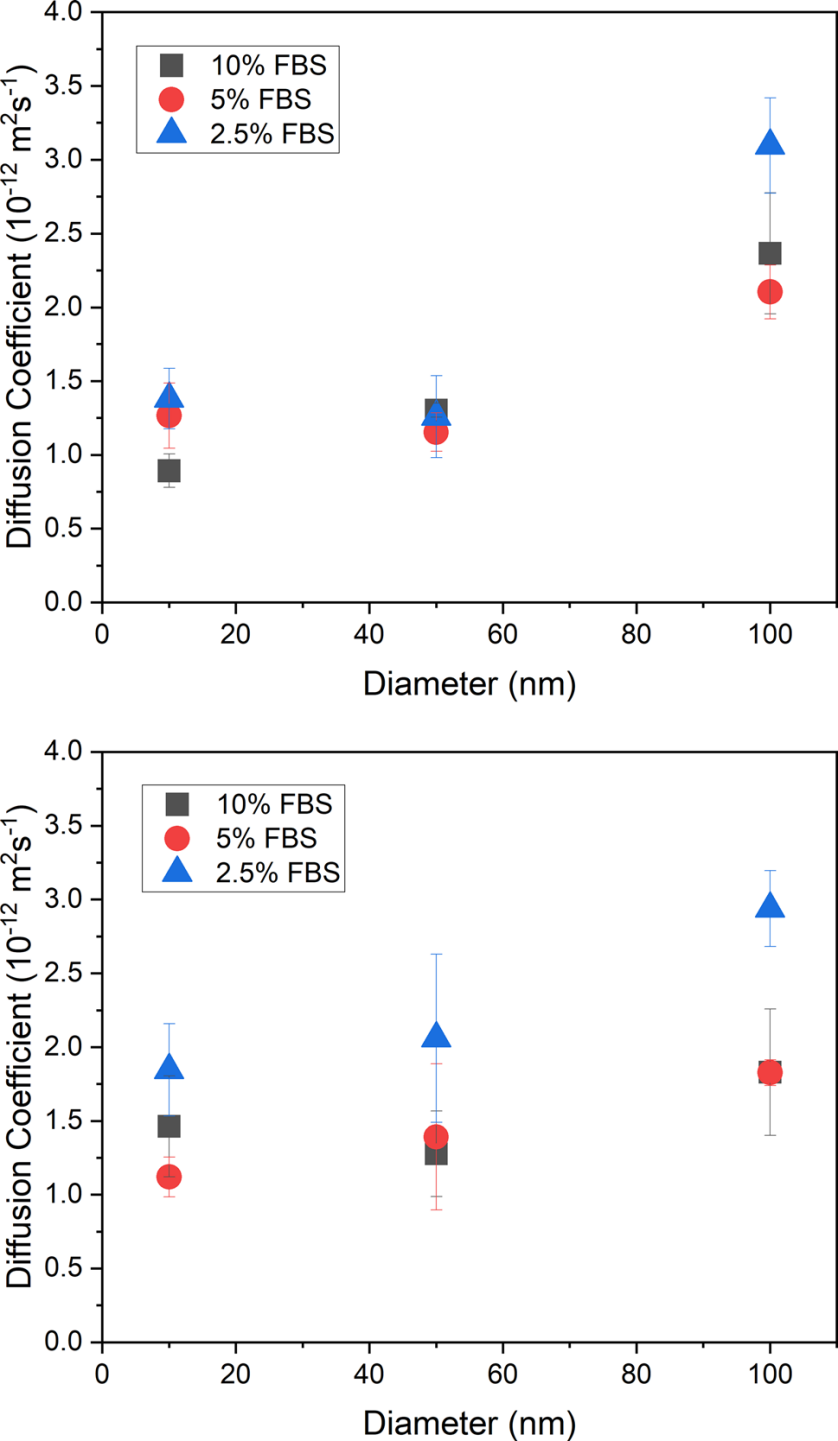
Average values (N = 4) of the diffusion coefficient for negatively-charged (top) and positively-charged (bottom) gold nanoparticles in DMEM supplemented media with different concentrations of FBS with error bars of ± 1 standard deviation.

Figure 5 shows the average values of diffusion coefficient as a function of the diameter of the nanoparticles. Positively-charged nanoparticles in 2.5% and 5% FBS media display a weak positive trend in the diffusion coefficient as nanoparticle size increased. As a result, there is a significant difference in the diffusion coefficient of 10 nm and 100 nm particles (*p* = 0.0034) at these FBS concentrations. Positively -charged nanoparticles in 10% FBS media show no significant change. Several studies have found that NP size is a critical factor in determining the thickness, structure and composition of the protein corona^7,9,10^. In the study by Piella et al.^9^ gold colloidal citrate nanoparticles with diameters 3.5–150 nm were dispersed in DMEM supplemented with 10% FBS. It was found that as nanoparticle size increased the protein corona became thicker, more laminar and composed of multilayers of proteins. The protein corona structure would therefore be different for 10 nm particles, whose size is closer to that of albumin (hydrodynamic diameter of ~ 7 nm^41^) than for 100 nm nanoparticles which are much larger than most serum proteins (20–30 nm^36^). Negatively-charged 100 nm nanoparticles were also found to have a significantly greater diffusion coefficient compared to both the 10 nm and 50 nm particles in all FBS concentrations (2.5%: *p* = 0.00030; 5%: *p* = 0.00032; 10%: *p* = 0.0043). This contradicts the Stokes–Einstein diffusion relationship, which predicts a slower diffusion speed for larger particles, meaning fundamental principles of diffusion cannot be applied in biofluids without considering the influence of protein interactions. We recognise that conformational changes in the three-dimensional protein structure due to a gain in free energy, which induces a re-organisation of the adsorbed protein molecule^42^, can occur. Structural re-arrangement is said to be dependent on various nanoparticle characteristics such as curvature, surface area and surface chemistry^43^ or could be specific to individual nanoparticle-protein interactions^31^. This could potentially influence the result seen in Fig. 5; however, this is beyond the scope of this study. Dynamic light scattering (DLS) was found to give unreliable estimations of nanoparticle size after corona formation at the particle concentrations used in this study, which is in agreement with Giorgi et al.^44^ and highlights the additional need for accessible and reliable techniques that can characterise protein corona thickness on nanoparticles, in situ, without the need for fluorescent labelling of the protein or particle.

## Conclusions

The development of safe and effective nanotechnologies for clinical application relies on the ability to track the diffusion and interactions of nanoparticles in biologically-relevant environments. It has been demonstrated that nanoparticle dynamics can be quantified in increasingly complex solutions using the caustics technique. Positively- and negatively-charged gold nanoparticles were dispersed in Dulbecco’s modified eagle medium (DMEM) with concentrations of fetal bovine serum (FBS) commonly used in cell growth and tracked using an optical, label-free microscopy technique which revealed novel results. The presence of proteins causes a reduction in the rate of diffusion of both positively- and negatively-charged nanoparticles with diameters 10 nm and 100 nm. The proteins form a corona around the nanoparticle which enlarges its effective diameter and can cause a transition from molecular motion described by the fractional Stokes–Einstein relationship to particle motion described by the linear Stokes–Einstein relationship in which the rate of diffusion is slower for particles with larger diameters. This was seen particularly for the largest nanoparticles which are close to the threshold size and for positively-charged nanoparticles. It was found that nanoparticle size and charge influenced their diffusion through biological media which would need to be considered when carrying out *in-vitro* tests under the conditions described in this study. The results of this study strengthen the point that biological interactions, specifically protein corona formation, cannot be universally predicted and it is clear that models linking protein dynamics to diffusion for nanoparticles in biologically-relevant conditions are needed to further the development of nanotechnologies.

## Data Availability

The datasets generated and/or analysed during the current study are available in the DataCat repository at: https://datacat.liverpool.ac.uk/2592/
